# A Study of Symmetry Breaking Predicates and Model Counting

**DOI:** 10.1007/978-3-030-45190-5_7

**Published:** 2020-03-13

**Authors:** Wenxi Wang, Muhammad Usman, Alyas Almaawi, Kaiyuan Wang, Kuldeep S. Meel, Sarfraz Khurshid

**Affiliations:** 8 Johannes Kepler University, Linz, Austria; 9 University of Birmingham, Birmingham, UK; 10 University of Texas at Austin, Austin, TX USA; 11 Google Inc., Sunnyvale, CA USA; 12 National University of Singapore, Singapore, Singapore

## Abstract

Propositional model counting is a classic problem that has recently witnessed many technical advances and novel applications. While the basic model counting problem requires computing the number of all solutions to the given formula, in some important application scenarios, the desired count is not of *all* solutions, but instead, of all *unique solutions up to isomorphism*. In such a scenario, the user herself must try to either use the full count that the model counter returns to compute the count up to isomorphism, or ensure that the input formula to the model counter adequately captures the *symmetry breaking predicates* so it can directly report the count she desires.

We study the use of *CNF-level* and *domain-level* symmetry breaking predicates in the context of the state-of-the-art in model counting, specifically the leading approximate model counter ApproxMC and the recently introduced exact model counter ProjMC. As benchmarks, we use a range of problems, including structurally complex specifications of software systems and constraint satisfaction problems. The results show that while it is sometimes feasible to compute the model counts up to isomorphism using the full counts that are computed by the model counters, doing so suffers from poor scalability. The addition of symmetry breaking predicates substantially assists model counters. Domain-specific predicates are particularly useful, and in many cases can provide *full* symmetry breaking to enable highly efficient model counting up to isomorphism. We hope our study motivates new research on designing model counters that directly account for symmetries to facilitate further applications of model counting.

## 1 Introduction

Propositional model counting is the classic problem of counting the number of all solutions for the given formula in propositional logic. While the core problem is an integral part of complexity theory literature, advances in propositional satisfiability (SAT) solvers and other decision procedures in the last decade have led to much progress in tackling this problem in innovative ways [7, 9, 10, 15, 17, 31, 39, 40, 47, 49, 50, 56, 64]. These advances have fueled the application of model counters in various software verification and reliability domains, e.g., to perform probabilistic analyses [13, 26, 28], check and repair string manipulation code [9, 41], and estimate information leakage using quantified information flow [19, 44].

While the basic model counting problem requires computing the number of *all* solutions, in some important application scenarios, the desired count is not of all solutions, but instead, of all *unique solutions up to isomorphism*, i.e., *non-isomorphic* (also called *non-symmetric*) solutions. For example, consider the context of software *reliability analysis* [26] where a goal is to find the number of inputs that can lead to an assertion violation, or *bounded exhaustive testing* [14, 42, 62, 68] where the goal is to estimate the total number of inputs that exist for a certain bound on the input size to decide what bound to use to stay within the testing budget. The desired counts in these cases are of non-isomorphic inputs, which are *non-equivalent* with respect to behaviors that a program can have because two inputs that are equivalent (and possibly not identical) produce the same output [66]. As another example, consider computing the number of solutions to a *constraint satisfaction problem* (CSP) [45], e.g., the number of unique ways 8 queens can be arranged on a fixed chess board such that no queen is under attack [6]. Once again, one is typically interested in the number of non-symmetric solutions because the indistinguishability of queens implies that a user does not consider two solutions obtained by swapping positions of queens to be unique.

In such scenarios, the user has two basic options. One option is to compute the full count using the model counter, and then use mathematical reasoning about symmetries to project the full count to the desired count. Doing so is straightforward in some cases, e.g., if each solution consists of $n$ indistinguishable objects of the same type and the composition of each solution implies that each permutation of those $n$ objects leads to a distinct (albeit isomorphic) solution, dividing the full count by $n!$ gives the count for non-isomorphic solutions; doing so is however, not always easy, for example when different solutions have different number of objects that can be permuted to form non-identical solutions. The other option is to ensure the formula that is input to the model counter includes *symmetry breaking predicates* [20, 21], i.e., additional constraints that only allow canonical solutions from each isomorphism class, so the model counter can report the desired count.

Symmetry breaking predicates can be added using three basic approaches [29]. Perhaps the most common approach is to add them at the CNF-level by using an off-the-shelf tool [8, 23], which takes as input a CNF formula and creates symmetry breaking predicates for it. Another common approach is to create them at the problem domain level using a domain-specific tool [58], and then translate the formula and predicates together to CNF. A third approach is to add them *manually* at the problem domain level [38, 59], and then translate to CNF.

A goal of our work is to study what is the best way to add symmetry breaking predicates (if at all) to obtain *precise* counts of non-isomorphic solutions. We conduct the study in the context of the state-of-the-art in model counting, specifically the leading approximate model counter *ApproxMC* [16, 17, 52] and the recently introduced exact model counter *ProjMC* [40]. ApproxMC and

ProjMC embody very different algorithms for model counting and provide us a diverse set of tools for the study. ApproxMC employs novel approximation methods to efficiently predict highly accurate model counts with formal guarantees, and is now in its third generation (called ApproxMC3 [52]). ProjMC uses a recursive algorithm and employs a disjunctive decomposition method together with a search for disjoint components, and just had its first public release.

As benchmark formulas, we use a range of problems, including structurally complex specifications of software systems [34] and constraint satisfaction problems [45]. To create the benchmark formulas, we employ the Alloy toolset [34] and its Kodkod backend [58]. Alloy allows writing formulas in relational first order logic with transitive closure, and has been used in academia and industry for design and specification of systems [11, 18, 35, 37, 65, 67, 70] as well as for various forms of analyses of code [27, 32, 36, 42, 48, 69]. The Alloy analyzer translates Alloy formulas with respect to a *scope*, i.e., bound on the universe of discourse, into propositional logic to create CNF problems that are solved using off-the-shelf SAT solvers [25]. Alloy supports fully automatic (partial) symmetry breaking at the level of Alloy specifications [51, 57] by adapting Crawford's symmetry breaking predicates [20], which are *statically* added to the formula *before* the solvers solve it. Alloy provides an ideal vehicle for evaluating the different approaches to symmetry breaking that are our focus in this study.

Similar to other techniques that use CNF-based backends, the Alloy analyzer translates problems from a higher-level (Alloy) to a lower-level (CNF). This translation often introduces new boolean variables in the resulting formula, which are not essential for creating the CNF formula but are required for a compact (feasible) encoding in CNF [60]. As a result, the translated formula is *equisatisfiable* to the original formula but may not be *equivalent* to it, and hence it may be the case that the model count for the CNF formula is very different from the original formula. Several modern model counters [16, 40, 50] readily handle this case by providing support for *projected model counting* [10], i.e., computing the model count with respect to a *subset* of all the variables. For Alloy, the subset is the *primary variables*, i.e., all boolean variables that directly correspond to the variables in the Alloy specification.

For each benchmark formula $f$, we create three model counting problems using automatic tools: 1) $f$ with no symmetry breaking, which we create by setting Alloy's default symmetry breaking to *off*; 2) $f$ with symmetry breaking predicates added at the problem domain level, which we create by having Alloy's default symmetry breaking turned *on*; and 3) $f$ with symmetry breaking predicates added at the CNF level, which we create by first using Alloy to create a CNF formula with no domain-level symmetry breaking, and then using the BreakID [23] tool to add CNF-level symmetry breaking predicates using its default settings. In addition, for select benchmarks we create formulas with manually added domain-specific symmetry breaking predicates, which we write in Alloy following previous work [38].

The results show that while it is sometimes feasible to compute the model counts up to isomorphism using the full counts that are computed by the model counters, doing so suffers from poor scalability. The addition of symmetry breaking predicates substantially assists model counters, although it is a well-known feature in *SAT solving* supported by theory finding [46, 61]. Domain specific predicates are particularly effective, and in many cases, can provide *full* symmetry breaking to enable highly efficient model counting up to isomorphism. We were surprised by the extent of the impact. Since the addition of symmetry breaking predicates introduces new dependencies among the variables, we expected these dependencies to make the formula more complex and perhaps less amenable to efficient model counting. However, the sheer reduction in the number of solutions caused by symmetry breaking more than compensates for the additional logical complexity of the formula. In cases where it was possible to create *full* symmetry breaking predicates, the model count for the formula *with* the predicates was computed up to a few orders of magnitude faster than the formula with *no* symmetry breaking predicates.

A key lesson of our study (in the context of the model counting problems considered) is: *if non-isomorphic solution counts are desired, use full symmetry breaking predicates at the domain-level whenever feasible – even if it is straightforward to compute the number of non-isomorphic solutions from the number of all solutions, or even if the symmetry breaking constraints have to be written manually.* This paper makes the following contributions:

- **Study.** To the best of our knowledge, we present the first study of symmetry breaking in the context of model counting. As pointed out earlier, there is a tradeoff between the reduction of solution space and the likely increase in complexity due to added symmetry breaking predicates. In prior work, the benefit of symmetry breaking in SAT solving were typically observed largely for *unsatisfiable* problems [43], our study shows the importance of symmetry breaking and its deep relation to problem formulation in the context of *satisfiable* problems, albeit for model counting.
- **Dataset.** All CNF files we used for the experiments are being made publicly available: `https://github.com/wenxiwang/TACAS2020`. We expect the dataset to be useful for future work on evaluating the performance of different model counters, and of the different strategies they employ, as well as for evaluating model enumeration tools.

We believe there is an important *bi-directional* relation between symmetry breaking and model counting whereas: 1) in one direction the model counters directly support computing the counts for non-isomorphic solutions to facilitate applications that so require; and 2) in the other direction symmetry breaking helps model counters become more efficient. We hope our study motivates future work that further investigates this relation.

## 2 Examples

This section provides two illustrative examples that require computing the number of unique solutions up to isomorphism. We specify the examples in the Alloy

```
module nqueens -- name of the specification sig Queen {} -- set of queen atoms one sig Board { state: Queen -> Int -> Int } -- one board fact StateOkay {
  all q: Queen | one q.(Board.state) -- each queen occupies exactly one cell
  all x: Queen.(Board.state).Int | ValidIndex[x] -- all x-coordinates are valid
  all y: Int.(Queen.(Board.state)) | ValidIndex[y] -- all y-coordinates are valid
  all disj q, r: Queen | q.(Board.state) != r.(Board.state) } -- queens do not share cells pred ValidIndex[x: Int] { x.gte[0] and x.lte[(#Queen).minus[1]] } -- x >= 0 && x <= |Queen|-1

fun X[q: Queen]: Int { (q.(Board.state)).Int } -- x-coordinate of q fun Y[q: Queen]: Int { Int.(q.(Board.state)) } -- y-coordinate of q fun Abs[x: Int]: Int { x.lt[0] implies negate[x] else x } -- absolute value of x pred SameRow[q, r: Queen] { X[q] = X[r] } -- q and r are in the same row pred SameColumn[q, r: Queen] { Y[q] = Y[r] } -- q and r are in the same column pred SameDiagonal[q, r: Queen] { -- q and r share a diagonal
  Abs[X[q].minus[X[r]]] = Abs[Y[q].minus[Y[r]]] }

pred NQueensProblem { -- no queen attacks another queen
   all disj q, r: Queen | !SameRow[q, r] and !SameColumn[q, r] and !SameDiagonal[q, r] }
```

Fig. 1: Alloy specification of $n$-Queens.

language, which allows us to explore different approaches for applying symmetry breaking. We provide intuitive descriptions of Alloy constructs as we introduce them; further details can be found elsewhere [34].

The first example illustrates a CSP problem [45] where Alloy's default symmetry breaking provides *full* symmetry breaking; we use ApproxMC to solve this problem (Section 2.1). The second example illustrates a software testing problem [42] where manually written symmetry breaking predicates provide full symmetry breaking; we use ProjMC to solve this problem (Section 2.2). Section 5 presents a detailed experimental evaluation where we use the two tools against many additional benchmarks.

## 2.1 $n$-Queens

Consider specifying the well-known *n-Queens* problem of placing $n$ interchangeable queens[4] on a fixed $n \times n$ chess-board, and computing the number of solutions to the problem using a modern propositional model counter [16, 40, 50].

Figure 1 shows a fragment of an Alloy specification of the $n$-Queens problem, which has been studied before using Alloy [2, 4, 55]. The keyword `sig` introduces a set of (interchangeable) atoms. The keyword `one` makes the set a singleton. The field `state` introduces a quaternary relation of type "`Board x Queen x Int x Int`" where `Int` is a built-in type that represents integers. The *fact* `StateOkay` describes the basic constraints for the state of the board to be valid; the fact contains

[4] Here, we only consider symmetries based on permuting the queens (and not other forms, e.g., rotations of the board.)

```
Queen={Queen$0, Queen$1, Queen$2, Queen$3,
       Queen$4, Queen$5, Queen$6, Queen$7}

Board={Board$0}

Board<:state={Board$0->Queen$0->7->5, Board$0->Queen$1->6->0,
              Board$0->Queen$2->5->4, Board$0->Queen$3->4->1,
              Board$0->Queen$4->3->7, Board$0->Queen$5->2->2,
              Board$0->Queen$6->1->6, Board$0->Queen$7->0->3}
```

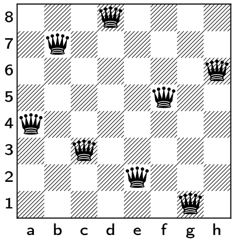

Fig. 2: A solution to 8-queens created by the Alloy analyzer illustrated.

4 sub-formulas that are implicitly conjoined; each of them uses universal quantification (`all`); the keyword `disj` constrains the quantified variables to represent distinct values. The dot operator ('`.`') is *relational join* [34]. A *predicate* (`pred`) is a parameterized formula that can be invoked elsewhere; likewise, a `fun` is a parameterized expression. The predicate `NQueensProblem` represents the overall specification of the $n$-Queens constraints. Any model of the Alloy specification must satisfy the constraints in all the facts and any predicates that are invoked (directly or transitively).

The Alloy user writes a *command* and executes it to solve desired constraints. For example, "`run NQueensProblem for 5 int, exactly 8 Queen`" asks the analyzer to find a solution to the 8-Queens problem. This command creates a constraint solving problem such that the integer bit-width is 5, and there are exactly 8 queens. Figure 2 shows a *valuation* for each set and relation created by the Alloy analyzer to solve this problem, and graphically illustrates the solution.

Next, we illustrate the use of the approximate model counter ApproxMC [16]. For the `nqueens` specification, for each $7 \leq n \leq 12$, we create three constraint solving problems: 1) no symmetry breaking (*no-sb*); 2) BreakID's default CNF-level symmetry breaking [23] (*cnf-sb*); and 3) Alloy's default domain-level symmetry breaking [58] (*dom-sb*). Table 1 shows the number of solutions found and time taken in each case. The model count with no symmetry breaking is the highest and takes the longest to compute; this approach times out for $8 \times 8$ and larger boards. BreakID's default CNF-level symmetry breaking significantly reduces

Table 1: ApproxMC results for $n$-Queens for $7 \leq n \leq 12$. Model count ("#") and time taken in seconds ("$t[s]$") for different problem sizes are shown. Time-out (t.o.) is 5000 sec.

| | | $7 \times 7$ | | $8 \times 8$ | | $9 \times 9$ | | $10 \times 10$ | | $11 \times 11$ | | $12 \times 12$ | |
|---|---|---|---|---|---|---|---|---|---|---|---|---|---|
| | | # | $t[s]$ | # | $t[s]$ | # | $t[s]$ | # | $t[s]$ | # | $t[s]$ | # | $t[s]$ |
| *approx* | *no-sb* | 208896 | 3727.1 | - | t.o. | - | t.o. | - | t.o. | - | t.o. | - | t.o |
| | *cnf-sb* | 67584 | 1446.4 | - | t.o. | - | t.o. | - | t.o. | - | t.o. | - | t.o |
| | *dom-sb* | 40 | 1.14 | 92 | 13.67 | 304 | 16.27 | 784 | 44.97 | 2752 | 199.77 | 15360 | 822.14 |
| *OEIS* | | 40 | | 92 | | 352 | | 724 | | 2680 | | 14200 | |
| *error* | | 0 | | 0 | | 0.158 | | -0.077 | | -0.026 | | 0.076 | |

the counts and the time. Alloy's default domain-level symmetry breaking is the most effective, and for this problem, removes all symmetries. Some of the approximate model counts reported by ApproxMC are coincidentally the *exact* counts. We validated the counts using the On-line Encyclopedia of Integer Sequences (OEIS) [6]: the sequence $\#A000170$ represents the number of solutions for the n-Queen problem. The counts computed using Alloy's default symmetry breaking with ApproxMC up to board size $8 \times 8$ form a subsequence of $A000170$. For the other board sizes, the table lists the error, which is $max(\frac{approx}{exact}, \frac{exact}{approx}) - 1$, based on multiplicative guarantees.

Note that the non-isomorphic solution count can easily be estimated from the full count for this problem. For example, for the $7 \times 7$ board we can estimate it as $\frac{208896}{7!} = 41.44$, which is quite close to the actual count of 40. While the calculation is simple, the time to compute the full count is much higher (3727.1 seconds instead of 1.14 seconds). Moreover, for larger board sizes, computing the full count times out, so using it for those sizes may be simply infeasible. This example illustrates a case where symmetry breaking predicates reduce both the model count and the time to compute it by relatively large factors.

**3-queens**. Table 2 shows the results for a variation of the $n$-queens problem where the number of queens is fixed to 3, and the board size varies. To specify this variation, we replace the expression "`(#Queen).minus[1]`" in predicate *ValidIndex* with the value of "$k - 1$" for the board size $k \times k$, and set the scope for Queen to "`exactly 3`" in the run command. We validate the ApproxMC counts using the OEIS sequence $\#A047659$ [6]. Once again, BreakID's CNF-level predicates significantly reduce the model count and time to compute it, and Alloy's domain-level predicates reduce them further. Since the number of queens is fixed to 3, the ratio of total number of solutions (*no-sb*) to number of non-isomorphic solution is $3! = 6$. For example, for $11 \times 11$ board, the ratio for ApproxMC counts is exactly 6; however, the time to compute the full count is, as before, much higher (1307.04 seconds instead of 45.1 seconds). This example shows a case where symmetry breaking predicates reduce the model count by a relatively small factor but the time to compute the counts by a much larger factor.

## 2.2 Data structure invariants

Next, consider the context of bounded exhaustive testing where the program under test is run against every non-equivalent input within a bound on the

Table 2: ApproxMC results for 3-Queens where 3 queens are placed on $n \times n$ board for $8 \leq n \leq 12$.

| | | $8 \times 8$ | | $9 \times 9$ | | $10 \times 10$ | | $11 \times 11$ | | $12 \times 12$ | |
|---|---|---|---|---|---|---|---|---|---|---|---|
| | | # | $t[s]$ | # | $t[s]$ | # | $t[s]$ | # | $t[s]$ | # | $t[s]$ |
| *approx* | *no-sb* | 64512 | 107.56 | 176128 | 368.65 | 335872 | 695.55 | 688128 | 1307.04 | 1081344 | 4811.86 |
| | *cnf-sb* | 18944 | 30.26 | 51200 | 67.43 | 122880 | 153.16 | 241664 | 280.15 | 417792 | 567.48 |
| | *dom-sb* | 9728 | 7.94 | 25088 | 12.78 | 57344 | 26.14 | 114688 | 45.1 | 200704 | 111.76 |
| *OEIS* | | 10320 | | 25096 | | 54400 | | 107880 | | 199400 | |
| *error* | | 0.061 | | 0.000 | | -0.051 | | -0.059 | | -0.006 | |

```
one sig BT {
  root: lone Node }

sig Node {
  left, right: lone Node }

pred Acyclic(t: BT) {
  all n: t.root.*(left + right) {
    n !in n.^(left + right) -- no directed cycle
    lone n.~(left + right) -- at most one parent
    no n.left & n.right }} -- children are different pred RepOk(t: BT) { Acyclic[t] }
...
```

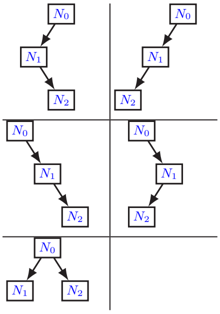

Fig. 3: (a) Alloy specification of binary trees. (b) Five non-isomorphic binary trees with 3 nodes. $N_0$ is the root.

input size, and the inputs are characterized by a logical formula [42]. Assume the goal is to identify a bound that will lead to a feasible number of inputs that can be executed within the testing budget. We use model counting to estimate the number of solutions for different bounds.

Assume the inputs to the program under test are *binary trees*. Figure 3a shows a partial Alloy specification for binary trees. The singleton sig `BT` represents the tree, which has a root node and an integer size; the keyword `lone` defines a partial function, so, e.g., the tree root is either exactly one node or none. Each node has an integer key and a left and a right child. The predicate `RepOk` specifies the constraints for a valid binary tree, which must be acyclic. The predicate `Acyclic` specifies acyclicity; the operator "`^`" is transitive closure, "`*`" is reflexive transitive closure, "`+`" is set union, "`&`" is set intersection, and "`~`" is transpose.

Consider the constraint solving problem for size $k$ so that the binary tree has exactly $k$ nodes and the keys are $1, \ldots, k$. Figure 3b illustrates the 5 non-isomorphic trees for size 3.

To show that the impact of symmetry is not limited to only approximate counting, we perform this case study with the exact model counter ProjMC [40]. Table 3 shows the model counts for different sizes. As before, CNF-level symmetry breaking reduces the model count, which is further reduced by Alloy's

Table 3: ProjMC results for binary tree constraints for trees with 6, 7, 8, 9, and 10 nodes. Time-out (t.o.) is 5000 sec.

| | | 6 | | 7 | | 8 | | 9 | | 10 | |
|---|---|---|---|---|---|---|---|---|---|---|---|
| | | # | $t[s]$ | # | $t[s]$ | # | $t[s]$ | # | $t[s]$ | # | $t[s]$ |
| *exact* | *no-sb* | 95040 | 5.57 | 2162160 | 129.25 | 57657600 | 3673.89 | - | t.o. | - | t.o. |
| | *cnf-sb* | 61538 | 7.39 | 1538628 | 184.97 | 25955296 | 3466.19 | - | t.o. | - | t.o. |
| | *dom-sb* | 357 | 0.10 | 1866 | 0.70 | 10286 | 4.94 | 60616 | 40.21 | 373001 | 610.35 |
| | *man-sb* | 132 | 0.03 | 429 | 0.09 | 1430 | 0.34 | 4862 | 1.48 | 16796 | 10.53 |
| *OEIS* | | 132 | | 429 | | 1430 | | 4862 | | 16796 | |

```
fact SymmetryBreaking { // pre-order
  BT.root in first[]
  all n: BT.root.*(left + right) {
    some n.left implies n.left in next[n]
    no n.left implies n.right in next[n]
    some n.right and some n.left implies
      n.right in next[max[n.left.*(left + right)]] }}
```

Fig. 4: *Full* symmetry breaking predicates in Alloy [38].

default symmetry breaking. However, unlike before, CNF-level symmetry breaking sometimes makes the model counter, which is ProjMC in this case, slower. Moreover, Alloy's default symmetry breaking does not break all symmetries. For this example, they can be broken using *manually written* predicates. Binary trees belongs to a restricted class of data structures for which *full* symmetry breaking can be achieved by writing predicates in Alloy so that only the *canonical* solution from each isomorphism class is allowed [38]. Figure 4 shows a fact that embodies this approach. Intuitively, the fact requires that a *pre-order* traversal starting at the root visits the nodes in the same order as a *pre-defined* linear ordering of the nodes; the `ordering` *module* in Alloy allows defining a linear order. The manually written predicates provide the most efficient counting. In this example the count up to isomorphism can, once again, be computed from the full count but at a much higher computational cost. For example, for 8 nodes, the full count is 57657600, which divided by 8! is 1430, i.e., the count up to isomorphism, but ProjMC takes 3673 seconds to compute the full count whereas once the manual symmetry breaking predicates are added it takes 0.34 seconds. The number of binary trees with $n$ nodes is the OEIS sequence #A000108, which allows us to validate that the manually written predicates are indeed breaking all symmetries.

## 3 Background: Model counting

This section gives the relevant background on model counting, with a focus on projected and approximate model counting.

Let $\varphi$ be a Boolean formula in conjunctive normal form (CNF) over the variable set $X$. An assignment $\sigma$ of truth values to the variables in $\varphi$ is called solution of $\varphi$ if it makes $\varphi$ evaluate to true. We denote the set of all witnesses of $F$ by $R_F$. Given a set of variables $S \subseteq X$ and an assignment $\sigma$, we use $\sigma \downarrow S$ to denote the projection of $\sigma$ on $S$. Similarly, $R_{\varphi\downarrow S}$ denotes projection of $R_\varphi$ on $S$.

The *projected model counting problem* is to compute $|R_{\varphi\downarrow S}|$ for a given CNF formula $F$ and sampling set $S \subseteq X$. When $S = X$, the problem is referred to as model counting. A *probably approximately correct* (or PAC) counter is a probabilistic algorithm $\mathsf{ApproxCount}(\cdot,\cdot,\cdot,\cdot)$ that takes as inputs a formula $F$, a sampling set $S$, a tolerance $\varepsilon > 0$, and a confidence $1-\delta \in (0,1]$, and returns a count $c$ such that $Pr\Big[|R_{\varphi\downarrow S}|/(1+\varepsilon) \le c \le (1+\varepsilon)|R_{\varphi\downarrow S}|\Big] \ge 1-\delta$. For clarity, we omit mention of $S$ unless needed for a given context.

Projected Model counting is a fundamental problem in computer science with applications ranging from reliability of networks to information leakage. Valiant initiated complexity theoretic studies of model counting and showed that model counting is #P-hard [63]. The earliest practical approaches to model counting such as Relsat [12], were based on extending DPLL approaches. The advent of CDCL solvers led to the paradigm of combining conflict driven search with component caching leading to the development of solvers such as Cachet [49] and sharpSAT [56]. Furthermore, Darwiche and Marquis [22] pioneered a knowledge-compilation-based approach, relying on the static partitioning of the solution space, which led to development of c2d. The recent years have witnessed combination of CDCL and static approaches with solvers such as D4 and DSharp. Recently, Lagniez and Marquis proposed a recursive algorithm, called ProjMC [40], that exploits the disjunctive decomposition technique pioneered in earlier works to perform projected model counting. Concurrently, another approach, called Ganak [50], for projected model counting has been developed that provides *probabilistic exact* bounds via usage of universal hash functions. In this work, we focus on ProjMC due to its ability to provide exact counts and demonstrated scalability in comparison to other approaches.

The theoretical studies of approximation led to the introduction of PAC style, also referred to as $(\varepsilon, \delta)$, guarantees wherein the underlying algorithm returns an estimate within $(1 + \varepsilon)$ factor of the exact count with confidence at least $1 - \delta$. Stockmeyer [54] demonstrated that PAC guarantees can be achieved by a probabilistic polynomial Turing machine with access to NP oracle. The practical exploration of Stockmeyer's approach was pursued with Gomes et al with the development of MBound [31] and SampleCount [30]. Chakraborty, Meel, and Vardi proposed a scalable approximate counter, called ApproxMC, with formal $(\varepsilon, \delta)$ guarantees which seeks to combine the advances in SAT solving with design of efficient universal hash functions.

ApproxMC is now in its third generation, called ApproxMC3. The central idea behind ApproxMC is to employ universal hash functions, represented by randomly chosen XOR constraints, to partition the solution space into roughly equal small cells where every cell can be defined by the original constraints augmented with randomly chosen XOR constraints. ApproxMC invokes CryptoMinisat [53], a solver designed specifically for combination of CNF and XOR constraints, to enumerate solutions in a randomly chosen *small* cell. ApproxMC2 achieves a significant reduction in the number of SAT calls from linear in $|S|$ to $\log(|S|)$ by exploiting dependence among different SAT calls. Soos and Meel proposed ApproxMC3 by augmenting ApproxMC2 with a new architecture to handle CNF+XOR formulas [52].

## 4 Study methodology

This section describes the overall design of our study, including the model counting tools, the generation of constraint solving problems, and the measurements for evaluation.

### 4.1 Tools

For approximate model counting, we use ApproxMCv3 (`https://github.com/meelgroup/ApproxMC`), which is the latest public release of ApproxMC [52]. For each model counting problem, we list the primary variables in the input CNF file as a comment as required by ApproxMC. For exact model counting, we use the latest public release of ProjMC [40] (`http://www.cril.univ-artois.fr/kc/projmc.html`). For each model counting problem, we list the primary variables in a separate file as required by ProjMC.

### 4.2 Benchmarks

**Base formulas**. We use four sources of base formulas.

(1) *Alloy specs.* We consider all Alloy specifications in the standard distribution [1]; each command in an Alloy spec defines a constraint solving problem and provides a scope; we use the given scope. We remove unsatisfiable problems since their model count is 0 (regardless of symmetry breaking), and our focus in this study is on satisfiable problems. We also remove all "easy" cases that complete within 1 second for both tools and all symmetry settings. This creates a set of 47 base problems derived from Alloy specifications.

(2) *Kodkod problems.* We consider all Kodkod programs in the standard distribution [5]. Once again, we remove the unsatisfiable problems and "easy" cases. In addition, we remove problems that do not admit symmetry breaking, i.e., where Kodkod does not add any symmetry breaking by default (e.g., when there is a given partial solution, which prevents Kodkod's *greedy base partitioning* [57] from having an effect). Some of the Kodkod programs are parameterized over integer bounds and input files. We manually create those inputs in the appropriate format. This gives us a total of 13 base problems derived from Kodkod programs.

(3) n-*Queens.* We use 2 common variations of the $n$-Queens problem: 1) $k$ queens are placed on a $k \times k$ board ($1 \leq k \leq 12$); 2) 3 queens are placed on a $k \times k$ board ($1 \leq k \leq 12$). This gives us a total of 24 base problems derived from the $n$-Queens problem[5].

(4) *Complex data structures.* We use 6 complex data structures: (1) singly-linked lists; (2) sorted lists; (3) doubly-linked lists; (4) binary trees; (5) binary search trees; and (6) red-black trees. For each structure, we bound the number of nodes to be between 6 and 9 (inclusive). This gives us a total of 24 base problems based on structural invariants.

**Model counting benchmarks**. For each base formula $f$, we create 3 model counting problems using automatic tools: 1) $f$ with no symmetry breaking, which we create by setting Alloy's default symmetry breaking to *off*; 2) $f$ with symmetry breaking predicates added at the CNF level, which we create by first using Alloy to create a CNF formula with no domain-level symmetry breaking, and then using the BreakID [23] tool to add CNF-level symmetry breaking predicates using the same arguments as in the SATRACE'15 competition [3]; and 3) $f$ with symmetry breaking predicates added at the problem domain level, which we create by having Alloy's default symmetry breaking turned *on.* Moreover, for data

[5] Unfortunately, we were not able to get the results for majority of the $n$-Queens benchmarks with ProjMC due to an unknown issue with the tool, so we do not use the $n$-Queens benchmarks for experiments with ProjMC; we have requested the ProjMC team to look into the issue.

structures, we create formulas with manually added domain-specific symmetry breaking predicates, which we write in Alloy following previous work [38]. This gives us a total of 348 model counting problems.

Table 4 shows some characteristics of the benchmarks, specifically the minimum and maximum numbers of primary variables, and all variables and clauses under the different symmetry breaking settings.

### 4.3 Metrics

We use two key metrics – the model counts and the time to compute them – and measure them under different symmetry breaking settings. For model counts, we report the tool output and the ratio of the count under one setting to the count under another setting. For time, we report the actual wall-clock times, and the ratio of time taken under one setting to the time taken under another setting. In line with prior work [17], we report the error rate of the approximate model counting which is $max(\frac{approx}{exact}, \frac{exact}{approx}) - 1$, based on multiplicative guarantees.

## 5 Experimental evaluation

The section reports the results of the experimental evaluation. Section 5.1 describes the results for ApproxMC. Section 5.2 describes the results for ProjMC.

### 5.1 Symmetry breaking and approximate model counting

**Time**. Figures 5a, 5c, and 5e illustrate the time performance of ApproxMC on the benchmarks based on Alloy, Kodkod, and data structure invariants respectively. With no symmetry breaking, ApproxMC times out on 21 (of 47) Alloy benchmarks, 6 (of 13) Kodkod benchmarks, and 10 (of 24) data structure benchmarks. In all but 16 cases, formulas with Alloy's default symmetry breaking take less time than with CNF-level symmetry breaking. In all but 10 cases, formulas with CNF-level symmetry breaking take less time than with no symmetry breaking. Moreover, for data structure benchmarks, in all but 1 cases, formulas with manual symmetry breaking take less time than Alloy's default symmetry breaking. Among all the problems that time out with no symmetry breaking, the smallest time taken by the corresponding problem with Alloy's default symmetry breaking was 0.14 seconds, and the smallest time taken by

Table 4: Benchmark characteristics.

| *source* | *#prim.* | *no-sb* | | *cnf-sb* | | *dom-sb* | | *man-sb* | |
|---|---|---|---|---|---|---|---|---|---|
| | | *#var.* | *#clause* | *#var.* | *#clause* | *#var.* | *#clause* | *#var.* | *#clause* |
| Alloy: min | 46 | 384 | 620 | 522 | 1037 | 384 | 620 | - | - |
| Alloy: max | 2048 | 93764 | 291349 | 93764 | 289725 | 93764 | 291349 | - | - |
| Kodkod: min | 48 | 631 | 188 | 932 | 628 | 990 | 188 | - | - |
| Kodkod: max | 8188 | 388755 | 764957 | 397566 | 834629 | 453358 | 877429 | - | - |
| n-Queens: min | 1024 | 3762 | 7163 | 3762 | 7163 | 3762 | 7163 | - | - |
| n-Queens: max | 12288 | 200074 | 532527 | 201064 | 523947 | 269141 | 704396 | - | - |
| Data Str.: min | 43 | 992 | 3039 | 1091 | 3337 | 1209 | 3401 | 1006 | 3155 |
| Data Str.: max | 510 | 18694 | 48290 | 19045 | 45562 | 19808 | 50212 | 18993 | 50696 |

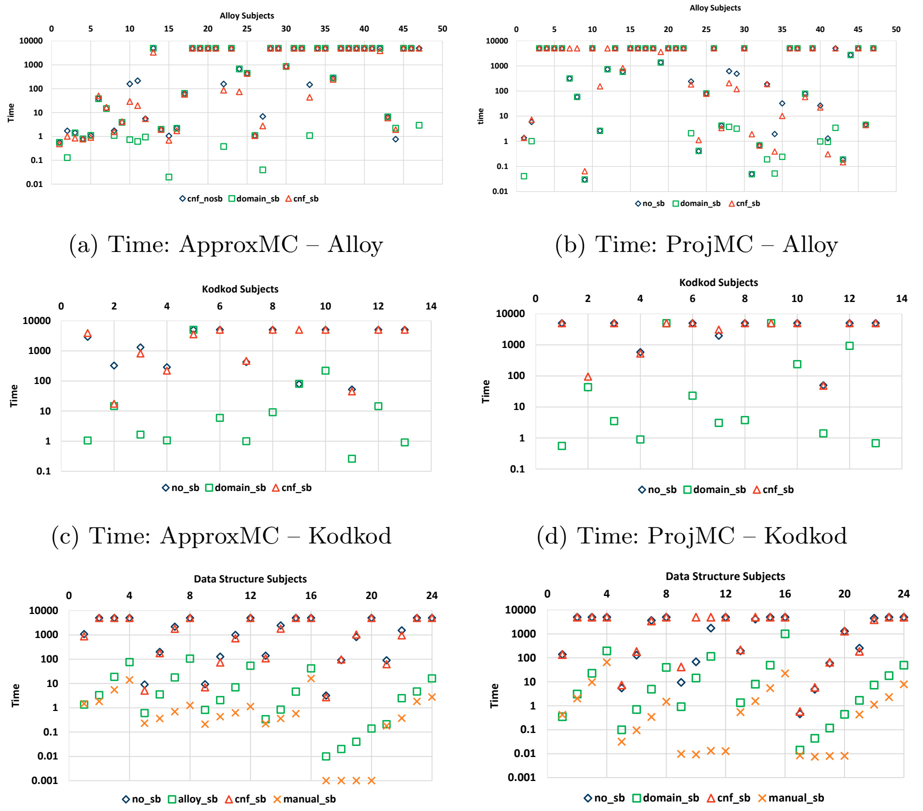

(a) Time: ApproxMC – Alloy

(b) Time: ProjMC – Alloy

(c) Time: ApproxMC – Kodkod

(d) Time: ProjMC – Kodkod

(e) Time: ApproxMC – Data structures

(f) Time: ProjMC – Data structures

Fig. 5: Time results. $x$-axis has benchmark model counting problems. $y$-axis has time in seconds (log-scale). Benchmarks on $x$-axis are sorted in ascending order based on the number of primary variables; moreover, the data structure benchmarks are grouped by the type of the structure. Blue diamond is no symmetry breaking (*no-sb*) ; red triangle is CNF-level symmetry breaking (*cnf-sb*); green square is Alloy's default symmetry breaking (*dom-sb*); and orange cross is manual symmetry breaking (*man-sb*).

the corresponding problem with manual symmetry breaking was 0.008 seconds. For the Alloy benchmarks, ApproxMC does not time-out under any symmetry breaking setting for benchmarks that have up to 90 primary variables. The time results for the $n$-Queens benchmarks were presented in Section 2.1.

**Model counts**. Figure 6a graphically illustrates how the model counts vary under different symmetry breaking settings. For the Alloy and Kodkod benchmarks, in all but 10 cases the model count for the formula with Alloy's default symmetry breaking is less than the corresponding count with CNF-level sym-

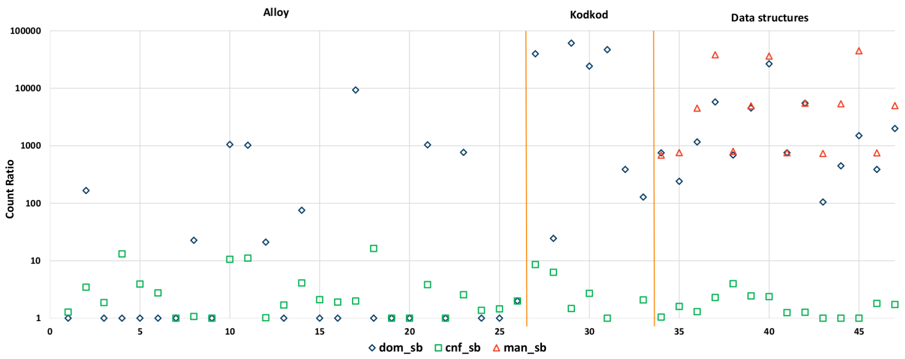

(a) Model count: ApproxMC

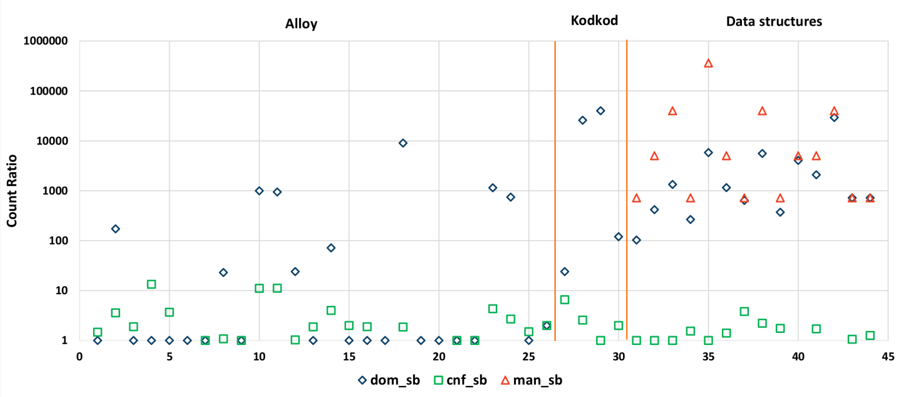

(b) Model count: ProjMC

Fig. 6: Model count results. $x$-axis has benchmark model counting problems. $y$-axis (log-scale) has count ratio $n/c$ where $n$ is the model count for the formula with no symmetry breaking and $c$ is the corresponding count with CNF-level symmetry breaking (green-square), Alloy's default symmetry breaking (blue-diamond), and manual symmetry breaking (red-triangle – only for data structures). Only cases where the calculation of $n$ did not time out are shown.

metry breaking. For the data structures, the model count for the formula with Alloy's symmetry breaking is less than the corresponding count with CNF-level symmetry breaking in all cases; moreover, in all but 5 cases, manual symmetry breaking gives the lowest count (the 5 exceptions are due to approximation in computing the model counts). Among all problems where ApproxMC reports a count with no symmetry breaking, the largest ratio of count with no symmetry breaking to count with Alloy's default symmetry breaking was 61167, and the largest ratio of count with no symmetry breaking to count with manual symmetry breaking was 45056. The model count results for the $n$-Queens benchmarks were presented in Section 2.1.

**Error**. For the Alloy, Kodkod, and data structure benchmarks, we compute the error in ApproxMC with respect to the counts reported by ProjMC for the cases where ProjMC reported a count. The error ranges were: [0, 0.168] for the Alloy benchmarks, [0, 0.168] for the Kodkod benchmarks, and [0, 0.165] for the data structure benchmarks. Section 2.1 presented the error results for the $n$-Queens benchmarks with respect to the number in OEIS [6].

### 5.2 Symmetry breaking and exact model counting

**Time**. Figures 5b, 5d, and 5f illustrate the time performance of ProjMC on the benchmarks based on Alloy, Kodkod, and data structure invariants respectively. With no symmetry breaking, ProjMC times out on 21 (of 47) Alloy benchmarks (which is the same number as ApproxMC although the two sets of benchmarks are not the same), 9 (of 13) Kodkod benchmarks (which is more that the number for ApproxMC), and 9 (of 24) data structure benchmarks (which is more than ApproxMC). In all but 8 cases, formulas with Alloy's default symmetry breaking take less time than with CNF-level symmetry breaking. In all but 24 cases, formulas with CNF-level symmetry breaking take less time than with no symmetry breaking. Moreover, for data structure benchmarks, in all but 2 cases, formulas with manual symmetry breaking take less time than Alloy's default symmetry breaking. Among all the problems that time out with no symmetry breaking, the smallest time taken by the corresponding problem with Alloy's default symmetry breaking was 3.12 seconds, and the smallest time taken by the corresponding problem with manual symmetry breaking was 0.01 seconds.
**Model counts**. Figure 6b graphically illustrates how the model counts vary under different symmetry breaking settings. For the Alloy and Kodkod benchmarks, in all but 9 cases the model count for the formula with Alloy's default symmetry breaking is less than the corresponding count with CNF-level symmetry breaking. For the data structures, the model count for the formula with Alloy's symmetry breaking is less than the corresponding count with CNF-level symmetry breaking in all cases; moreover, in all cases, manual symmetry breaking gives the lowest count. Among all problems where ApproxMC reports a count with no symmetry breaking, the largest ratio of count with no symmetry breaking to count with Alloy's default symmetry breaking was 40320, and the largest ratio of count with no symmetry breaking to count with manual symmetry breaking was 362880.

Overall, the impact of symmetry breaking is significant for both ApproxMC and ProjMC. In majority of the cases, Alloy's default symmetry breaking is more effective than CNF-level symmetry breaking using BreakID. For data structure benchmarks, manual symmetry breaking is the most effective, and reports exactly the counts of the non-isomorphic solutions as desired; moreover, in cases where Alloy's default symmetry breaking provides *full* symmetry breaking, manual symmetry breaking provides much faster solving.

### 5.3 Discussion

The empirical evaluation in the preceding subsections clearly demonstrates the significant impact of symmetry breaking on ApproxMC and ProjMC. While a detailed study to explain the observed behavior is beyond the scope of this work, we offer some explanations. As pointed out by Soos and Meel [52], over 99% of the runtime of ApproxMC is consumed by the underlying SAT solver handling CNF-XOR formulas. The usage of symmetry breaking predicates for satisfiable instances typically leads to smaller overheads in runtime in the context of satisfiability queries. As discussed above, the use of symmetry breaking predicates significantly reduces the number of solutions and thereby leads to the significant reduction in the number of XORs to be added by ApproxMC. Note that the number of XORs to be added is logarithmically proportional to the number of solutions of a formula. The performance of SAT solvers has been observed to be sensitive to the number of XORs [24] and therefore, we believe that reduction in the required number of XORs is the primary reason behind the performance improvements in the context of ApproxMC.

The performance improvement of ProjMC is, however, more surprising since it is not necessarily the case that reduction in the number of solutions would lead to reduction in the size of the corresponding d-DNNF (decision-Deterministic Decomposable Negation Normal Form), which represents the trace of the execution of ProjMC [33]. Furthermore, given the lack of noticable difference in runtime performance improvement via off-the-shelf symmetry breaking tools, it would be an interesting direction of future work to understand the difference in the traces between the formulas generated via Alloy's default symmetry breaking and CNF-level symmetry breaking.

## 6 Conclusions

This paper presented, to the best of our knowledge, the first study of symmetry breaking and model counting. A goal of the study was to determine what is the best way to add symmetry breaking predicates (if at all) to obtain precise counts of non-isomorphic solutions. We studied two model counters from two different classes and four scenarios of applying symmetry breaking. A key lesson of our study is that domain-specific symmetry breaking predicates are most effective at enabling precise computation of model counts up to isomorphism. We believe the results of our study can provide insights into more effective use of cutting edge model counters in important domains where the number of unique solutions up to isomorphism is desired, and also enable developing novel model counting methods that exploit symmetries.

## Acknowledgments

This work was supported in part by the U.S. National Science Foundation Grant CCF-1718903, and the National Research Foundation Singapore under its AI Singapore Programme [AISG-RP-2018-005].
